# CCM proteins control endothelial β1 integrin dependent response to shear stress

**DOI:** 10.1242/bio.201410132

**Published:** 2014-11-28

**Authors:** Zuzana Macek Jilkova, Justyna Lisowska, Sandra Manet, Claude Verdier, Valerie Deplano, Christian Geindreau, Eva Faurobert, Corinne Albigès-Rizo, Alain Duperray

**Affiliations:** 1 INSERM, Institut Albert Bonniot, F-38000 Grenoble, France; 2 Université Grenoble Alpes, Institut Albert Bonniot, F-38000 Grenoble, France; 3 CNRS ERL 5284, F-38042 Grenoble, France; 4 CNRS/Université Grenoble 1, LIPhy, UMR 5588, F-38041 Grenoble, France; 5 Aix-Marseille Université, CNRS, Centrale Marseille, IRPHE UMR 7342, F-13384, Marseille, France; 6 CNRS UMR5521, 3SR, Université Joseph Fourier Grenoble-INP, Grenoble, F-38042, France

**Keywords:** β1 integrin, CCM, Shear stress, Mechanotransduction

## Abstract

Hemodynamic shear stress from blood flow on the endothelium critically regulates vascular function in many physiological and pathological situations. Endothelial cells adapt to shear stress by remodeling their cytoskeletal components and subsequently by changing their shape and orientation. We demonstrate that β1 integrin activation is critically controlled during the mechanoresponse of endothelial cells to shear stress. Indeed, we show that overexpression of the CCM complex, an inhibitor of β1 integrin activation, blocks endothelial actin rearrangement and cell reorientation in response to shear stress similarly to β1 integrin silencing. Conversely, depletion of CCM2 protein leads to an elongated “shear-stress-like” phenotype even in the absence of flow. Taken together, our findings reveal the existence of a balance between positive extracellular and negative intracellular signals, i.e. shear stress and CCM complex, for the control of β1 integrin activation and subsequent adaptation of vascular endothelial cells to mechanostimulation by fluid shear stress.

## INTRODUCTION

In the vascular system, forces produced by blood flow and basement membrane are critical determinants of blood vessels physiological function and pathological states (Davies, 2009; Hahn and Schwartz, 2009; Chiu and Chien, 2011). Despite the fact that endothelial phenotypes are heterogeneous over the arterial tree, all endothelial cells (EC) are capable of responding to fluid wall shear stress by changing their orientation and shape together with modifying their distribution of cytoskeletal components (Dewey et al., 1981; Franke et al., 1984; Katoh et al., 2008). The best described is actin cytoskeleton remodeling in response to shear stress. This early response is characterized by actin filaments rearrangement into bundles of stress fibers and gradual reorientation parallel to the flow direction, which later results in cell alignment and cell shape optimization (Noria et al., 2004; Osborn et al., 2006), characteristic for healthy endothelium. On the other hand, disorganization of cell shape is one of the markers of endothelial dysfunction *in vivo* (Katoh et al., 2008; Chiu and Chien, 2011). EC adaptation to shear stress is the integrated response of signaling networks at different subcellular locations. One of the best-studied mechanotransducers is a complex of proteins at cell–cell junctions, consisting of platelet endothelial cell adhesion molecule 1 (PECAM-1) which transmits the hemodynamic signal to vascular-endothelial cadherin (VE-cadherin) and vascular endothelial growth factor receptor 2 (Tzima et al., 2005; Conway et al., 2013). This complex activates the production of phosphoinositides by PI3K which in turn activates integrins by an inside-out mechanism through the recruitment of activators to their cytoplasmic tail (Collins et al., 2012). Therefore, it appears that shear stress is an extracellular mechanical signal which triggers signaling pathways going from cell–cell to cell–Extracellular Matrix (ECM) adhesion and actin remodeling (Hahn and Schwartz, 2009).

Interestingly, cell–cell and cell–ECM adhesions are also coordinated by an intracellular node, the CCM complex comprising CCM1 and CCM2 (Faurobert and Albiges-Rizo, 2010; Draheim et al., 2014). It regulates VE-cadherin-dependent cell–cell interactions (Glading et al., 2007; Dejana and Orsenigo, 2013), β1 integrin-dependent cell–ECM adhesion (Faurobert et al., 2013) and acto-myosin remodeling (Whitehead et al., 2009; Stockton et al., 2010). The loss-of-function of CCM proteins in humans leads to Cerebral Cavernous Malformations corresponding to stacks of dilated blood vessels lacking mural cells and from where blood extravasates, damaging the adjacent neural tissue (Fischer et al., 2013; Draheim et al., 2014). These lesions form only in low blood-flow venous capillaries and the reason why is yet not known. CCM1/CCM2 complex controls inside-out β1 integrin activation by orchestrating the competition between the inhibitor ICAP-1 and the activators kindlin and talin for the binding to β1 integrin cytoplasmic tail (Millon-Frémillon et al., 2008; Brunner et al., 2011; Liu et al., 2013). We have shown that ICAP-1 protein is degraded when CCM1 or CCM2 are lost, and that this leads to overactivation of β1 integrin (Faurobert et al., 2013). As a consequence, RhoA/ROCK-dependent cell contractility increases the formation of transversal actin stress fibers (Whitehead et al., 2009; Stockton et al., 2010; Faurobert et al., 2013). Interestingly, β1 integrin has been involved in the response of EC to cyclic strain or shear stress (Urbich et al., 2000; Jalali et al., 2001; Thodeti et al., 2009). These results pinpoint to β1 integrin as an interesting target for the integrated regulation of EC response to shear stress.

Here, we demonstrate that the control of β1 integrin signaling is crucial for the shear-stress-induced response of EC and we provide a novel insight into the endothelial mechanotransduction pathways. We give clear evidence that an inhibition in β1 integrin signaling due to higher expression of CCM proteins is correlated with the failure of response to shear stress. We show that either shear stress or loss of CCM complex stimulates β1 integrin-dependent actin rearrangement in stress fibers, supporting a model in which β1 integrin activation is regulated by antagonistic extra- and intracellular signals, i.e. shear stress and CCM complex. It is likely that this balance is crucial for the homeostasis of low-flow venous capillaries in which CCM complex puts a cap on a deleterious β1-dependent cell response.

## RESULTS

### Defective β1 integrin activation is responsible for the inability of cells to orient under shear stress

**Fig. 1.**
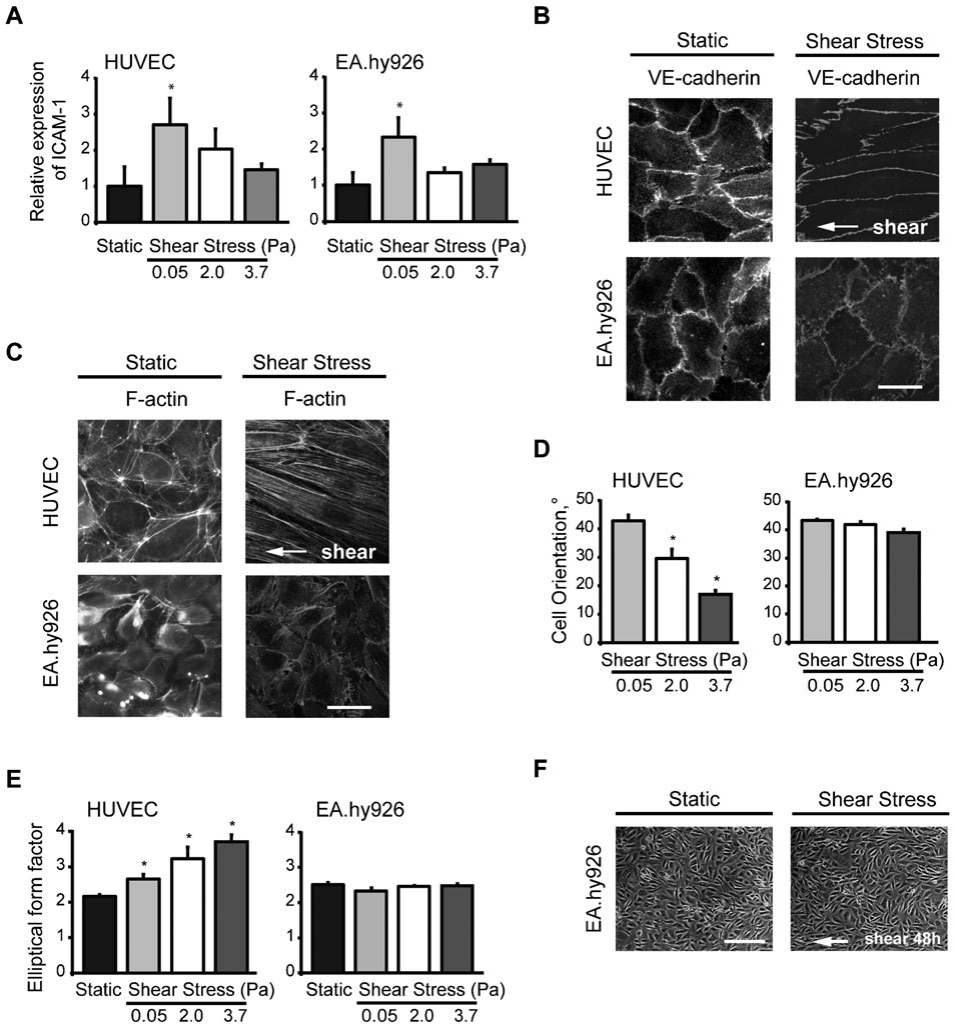
Contrary to HUVEC, EA.Hy926 cell line does not align in response to shear stress. (A) Cell surface expression of ICAM-1 on HUVEC and EA.hy926 exposed or not to shear stress of increasing strength for 18 h. Data are means ± SE (N = 5), *p<0.05, significantly different from static condition. (B) HUVEC and EA.hy926 cell line under static condition or exposed to fluid shear stress at 2.0 Pa for 5 h were stained by VE-cadherin. (C) The organization of the actin cytoskeleton of HUVEC and EA.hy926 monolayers under static conditions or after shear stress stimulation (2.0 Pa for 18 h). White arrow: indicates the direction of flow. (D) Alignment of cells was determined as cell orientation (0° indicates perfect alignment with flow direction; 45° indicates random orientation). (E) Cell elongation was established as the elliptical form factor (increasing levels of elliptical form factor indicates elongation). Data are means ± SE (N = 5), *p<0.05. (F) Confluent EA.hy926 monolayers were maintained under static conditions or exposed to shear stress of 3.7 Pa for 48 h. White arrow indicates flow direction. The results shown are representative of three independent experiments. Scale bars: 10 µm (B,C), 100 µm (F).

EC sense and transduce shear stress through multiple proteins, including VE-cadherin at cell–cell junctions and integrins at cell–matrix adhesions, resulting in diverse range of responses at different levels, as are modifications of expression of inflammatory proteins like ICAM-1 or changes in adhesion sites. We compared biological responses to hemodynamic shear stress between HUVEC and EA.hy926 cell line, a fusion of HUVEC with adenocarcinoma cells which is widely used as a convenient endothelial model in vascular research. As expected, ICAM-1 expression was increased in both cell lines upon low shear stress stimulation (Fig. 1A). This result is consistent with in vivo observations of artheroprone areas, characterized by low or disturbed flow, where EC show increase in the expression or activation of ICAM-1 compared to EC from regions of higher laminar shear stress (Endres et al., 1997; Nakashima et al., 1998; Iiyama et al., 1999; Suo et al., 2007).

Although HUVEC and EA.hy926 cell line both expressed VE-cadherin with typical location at the cell-to-cell adherens junctions under static condition same as after 18 h of shear stress of 2 Pa (Fig. 1B), they showed striking differences in actin cytoskeleton-remodeling in response to fluid shear stress. After 18 h of shear stress of 2 Pa, HUVEC displayed long and well-organized transversal actin stress fibers largely aligned with the direction of the flow whereas EA.hy926 cells subjected to shear stress were characterized by short actin filaments localized at the periphery of cells (Fig. 1C). Compared to static state, characterized by random orientation of cells, HUVEC progressively elongated and oriented in the direction of flow upon increasing shear hemodynamic forces (Fig. 1D,E). In contrast, EA.hy926 did not elongate nor re-orient even under high shear stress for 18 h (Fig. 1D,E) or after prolonged exposure (48 h) to high shear stress (Fig. 1F).

**Fig. 2.**
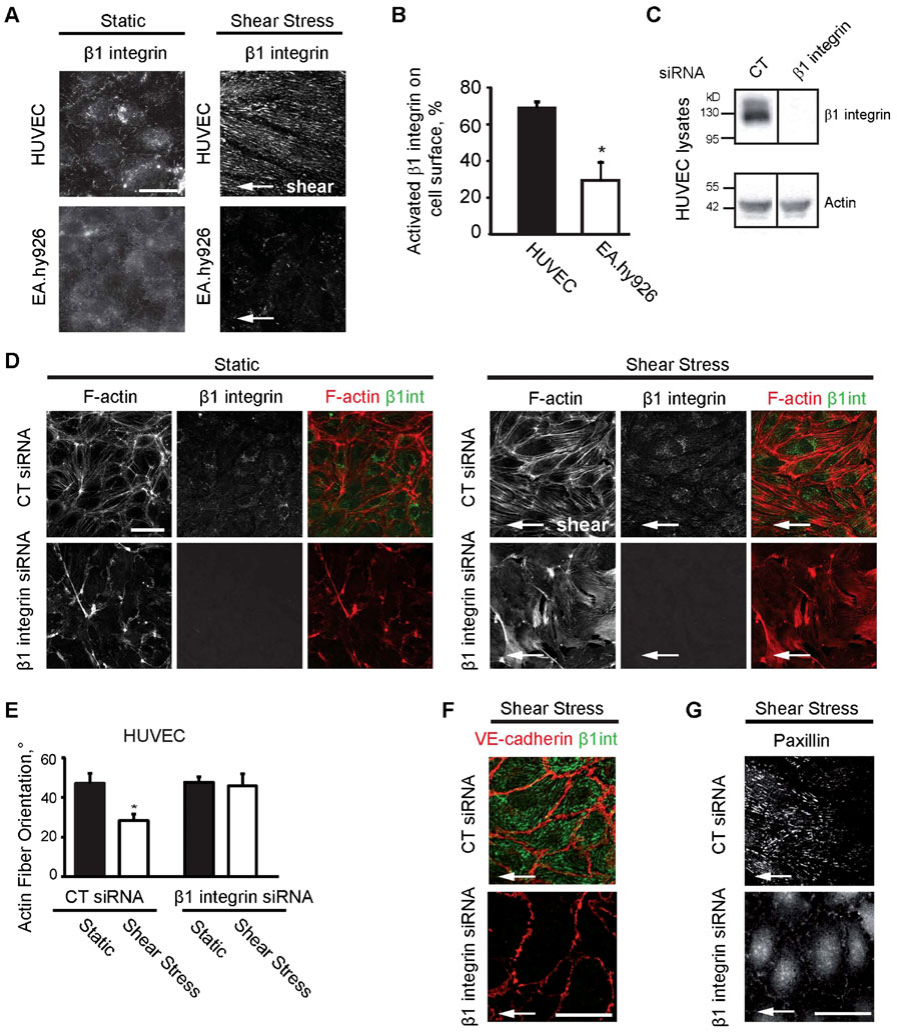
Impaired β1 integrin activation correlates with the inability of EC to respond to fluid shear stress. (A) HUVEC and EA.hy926 monolayers after static condition or fluid shear stress at 2.0 Pa for 18 h. White arrow: indicates the direction of flow. Cells were stained by activated-state-specific β1-integrin antibody (Clone:9EG7). (B) Percentage of activated β1 integrin in the total β1 integrin population on the surface of HUVEC and EA.hy926 cell line analyzed by flow cytometry. Data are means ± SE (N = 3), *p<0.05. (C) Silencing of β1 integrin by siRNA in HUVEC analyzed by western blot. (D) CT- or β1 integrin-silenced HUVEC were maintained under static conditions or exposed to fluid shear stress at 2.0 Pa for 5 h. F-actin and activated β1 integrin were stained. White arrow: indicates the direction of flow. (E) Actin stress fiber orientation (0° indicates perfect alignment with flow direction; 45° indicates random orientation). Data are means ± SE (N = 6), *p<0.05. CT- or β1 integrin-silenced HUVEC exposed to fluid shear stress at 2.0 Pa for 5 h were stained by (F) VE-cadherin and activated-state-specific β1-integrin antibody (Clone:9EG7) and (G) by paxillin antibody. Scale bars: 10 µm (A), 50 µm (D), 20 µm (F,G).

**Fig. 3.**
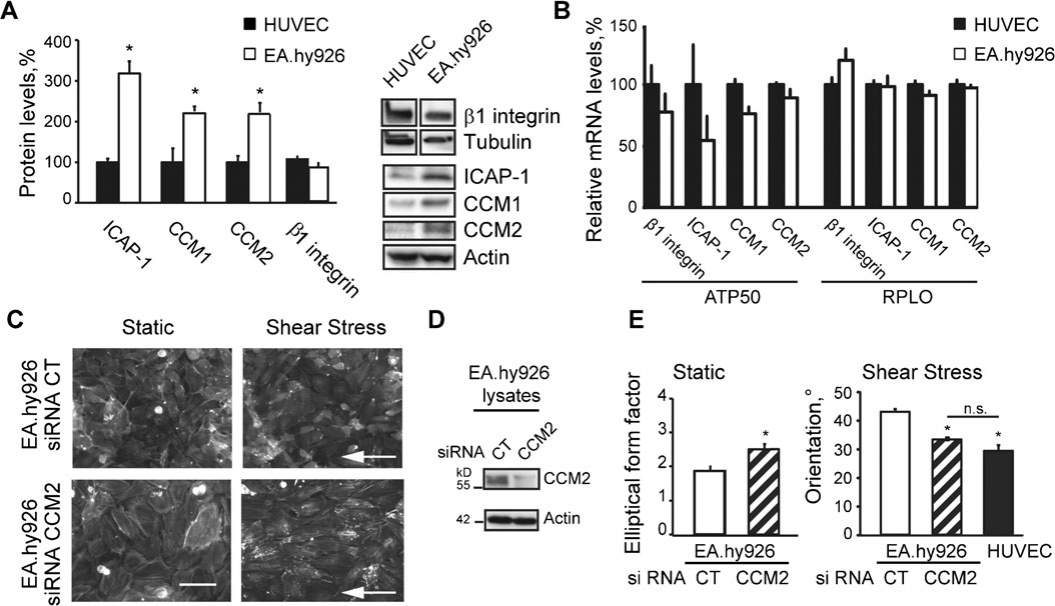
CCM complex inhibits β1 integrin-dependent morphological response of EC to fluid shear stress. (A) Western blots of β1 integrin protein, ICAP-1, CCM1 and CCM2. Quantifications are means ± SE (N = 8). (B) Relative mRNA levels of β1 integrin, ICAP-1, CCM1 and CCM2 in HUVEC (set as 100%) and in EA.hy926 cell line. Black bars, HUVEC; white bars, EA.hy926 cell line. Data are means ± SE (N = 4) normalized using housekeeping genes ATP 50 and RPLO. (C) Staining of F-actin on CT- or CCM2-silenced EA.hy926 under static conditions or exposed to fluid shear stress at 2.0 Pa for 18 h. White arrow: indicates the direction of flow. (D) Silencing of CCM2 by siRNA in EA.hy926 analyzed by western blot. (E) Elongation of CT- or CCM2-silenced EA.hy926 under static conditions and cell orientation of CT- and CCM2-silenced EA.hy926 or HUVEC exposed to fluid shear stress at 2.0 Pa for 18 h. Data are means ± SE (N = 2), *p<0.05. Scale bar: 25 µm (C).

As β1 integrin has a major role in organizing acto-myosin cytoskeleton in EC (Faurobert et al., 2013) and is upregulated (Urbich et al., 2000) and activated (Jalali et al., 2001) by shear stress, we monitored the behavior of β1 integrin in both cell lines. We observed that upon shear stress, activated β1 integrin (stained with activated-state-specific β1-integrin antibody) clustered into numerous adhesion sites in HUVEC underneath the actin stress fibers (Fig. 2A) whereas only rare β1 integrin-containing focal adhesions could be detected in EA.hy926 and their number did not increase upon shear stress (Fig. 2A). We next examined the status of β1 integrin on cell surface by flow cytometry using HUTS-4 antibody which binds specifically to β1 integrin activated form. Interestingly, the percentage of activated β1 integrin was 2.5-fold lower in EA.hy926 cell line than in HUVEC under static conditions (Fig. 2B) although the total amount of β1 integrin protein was similar (Fig. 3A). Therefore, we correlated the inability of EA.hy926 to activate β1 integrin and to form β1 integrin-containing focal adhesions with their inability to respond to shear stress.

To test the hypothesis that the defective β1 integrin signaling is responsible for the inability of EC to align and elongate under flow, we silenced β1 integrin in HUVEC and studied its effect on the response to shear stress. Silencing efficiency of β1 integrin was assessed by western blot (Fig. 2C). Since our preliminary results showed that β1 integrin depletion causes significant detachment of cells after 6 hours of shear stress exposure, which is not surprising considering the role of β1 integrin in cell adhesion, the time of shear stress stimulation was reduced to 5 hours. Therefore, instead of cell orientation, the actin fiber orientation as the early response to shear stress was determined. In fact, as was demonstrated previously, the cell alignment itself requires 18–24 h of exposure to flow, but the cells respond already within first hours by cytoplasmic actin-reorientation (Osborn et al., 2006). As anticipated, CT-silenced HUVEC had reoriented their actin filaments parallel to the flow, and were already partially elongated after 5 h of shear stress (Fig. 2D,E). On the contrary, β1 integrin-silenced HUVEC failed to rearrange their actin microfilaments and cells retained their polygonal shape and random orientation characteristic for static conditions (Fig. 2D,E). β1 integrin depletion had no major effect on VE-cadherin localization as demonstrated by its staining at cell–cell junctions either under static or upon shear stress (Fig. 2F). This suggests that the initial step of the mechanoresponse starting at cell–cell junctions should not be fundamentally perturbed by β1 integrin silencing. By contrast, upon shear stress, cell–ECM adhesion was strongly affected by β1 integrin-silencing. We observed delocalization of paxillin from β1 integrin-dependent focal adhesions to the cytoplasm and to peripheral focal adhesions, most likely dependent on other β integrins (Fig. 2G). Whereas the inability of the β1 integrin-depleted HUVEC to respond to shear stress was not caused by major defects in cell–cell junctions, our results rather show that β1 integrin-dependent signaling is necessary for EC alignment and elongation in response to shear stress.

### CCM complex controls β1 integrin-dependent morphological response of EC to shear stress

We have previously demonstrated that CCM complex, which is crucial in endothelial physiology, inhibits β1 integrin activation in EC (Faurobert et al., 2013). We thus wondered whether EA.hy926 express less activated β1 integrin on their surface and do not form β1 integrin-dependent actin stress fibers in response to shear stress because of abnormal expression of CCM complex. Strikingly, relative to actin levels, protein levels of ICAP1, CCM1 and CCM2 were significantly higher, between two and three folds, in EA.hy926 compared to HUVEC whereas the β1 integrin levels were similar (Fig. 3A). Post-transcriptional events were likely involved as no statistical difference in the level of mRNA coding for these proteins could be measured between HUVEC and EA.hy926 (Fig. 3B). Thus it appeared that the fusion of HUVEC with adenocarcinoma A549 cells has generated an endothelial cell line which overexpresses the ICAP-1/CCM complex.

**Fig. 4.**
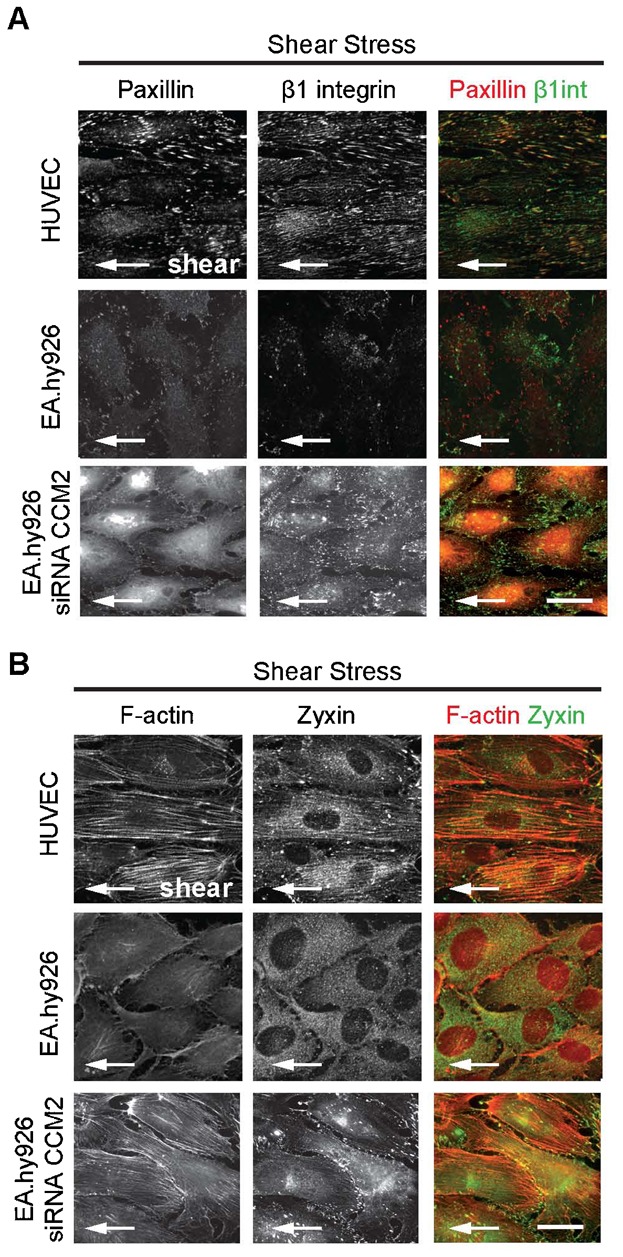
CCM2 negatively regulates β1-integrin mediated cytoskeletal reinforcement and reorientation after shear stress stimulation. HUVEC, EA.hy926 and CCM2-silenced EA.hy926 were exposed to fluid shear stress at 2.0 Pa for 18 h. Cells were stained by (A) paxillin and activated-state-specific β1-integrin antibody (Clone:9EG7) and by (B) F-actin and zyxin antibody. White arrow: indicates the direction of flow. Scale bars: 10 µm.

To investigate whether the high levels of CCM proteins are indeed the reason for the inability of EA.hy926 to respond to shear stress, we analyzed the response of CCM2-silenced EA.hy926 to shear stress. As we have previously shown, CCM2 silencing induces the destabilization of its partner proteins CCM1 and ICAP-1 resulting in the loss of the entire complex (Faurobert et al., 2013). Silencing efficiency of CCM2 siRNA on EA.hy926 cell line was determined by western blot (Fig. 3D). Remarkably, CCM2 deficiency led to elongation of the EA.hy926 cells (Fig. 3E) and restored their capacity to orient toward the flow (Fig. 3C,E) similarly to HUVEC. This was correlated with the appearance of aligned β1 integrin-containing focal adhesions on the ventral face of CCM2-depleted EA.hy926 (Fig. 4A). Paxillin was recruited to these focal adhesions (Fig. 4A) showing that release of β1 integrin inhibition upon CCM2 loss led to the generation of functional β1 integrin-dependent signaling scaffolds in response to shear stress. Transversal actin fibers were polymerized from these β1 integrin scaffolds and generated internal tension as suggested by the recruitment of zyxin (Fig. 4B), a typical component of focal adhesions which accumulates at force-bearing sites (Beningo et al., 2001; Colombelli et al., 2009) and is mobilized by mechanical forces from focal adhesions to actin filament (Yoshigi et al., 2005). Therefore, contrary to EA.hy926, shear stress increased internal cell tension in CCM2-depleted EA.hy926 in a similar way as it did in HUVEC. These results confirm that overexpression of CCM/ICAP-1 proteins is responsible for the absence of morphological response of EA.hy926 most likely by preventing shear-stress-induced β1 integrin activation.

**Fig. 5.**
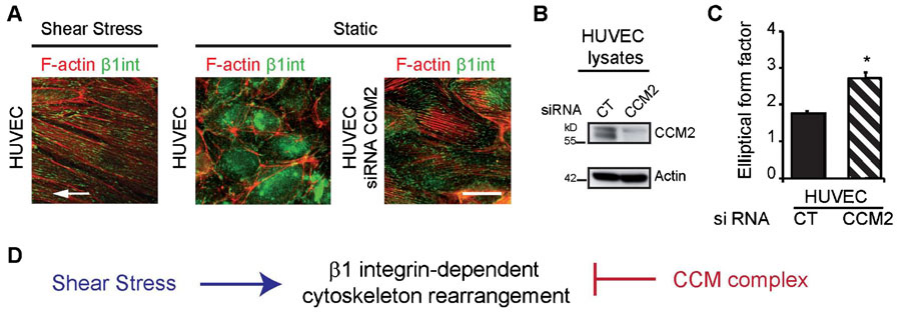
β1 integrin-dependent signaling is regulated by a balance between fluid shear stress and CCM complex. (A) Activated-state-specific β1-integrin and F-actin staining of HUVEC exposed to fluid shear stress at 2.0 Pa for 18 h or under static conditions and CCM2-silenced HUVEC under static conditions. White arrow: indicates the direction of flow. (B) Silencing of CCM2 by siRNA in HUVEC analyzed by western blot. (C) Elongation of CT- or CCM2-silenced HUVEC under static conditions. Data are means ± SE (N = 3), *p<0.05. (D) Shear stress and CCM complex act antagonistically on β1 integrin-dependent morphological response of EC. Scale bar: 10 µm (A).

Finally, we wondered whether depletion of CCM2 would lead to a “shear-stress-like” phenotype even without flow. We indeed confirmed that CCM2-silenced HUVEC (Fig. 5B) adopted an elongated shape associated with the formation of β1 integrin-anchored stress fibers in static condition, reminiscent of what is observed for naïve HUVEC under shear stress (Fig. 5A,C).

Overall, we showed that the mechanical response necessary for EC to cope with external hemodynamic forces depends on the control of β1 integrin activation by a positive extracellular signal, i.e. shear stress and a negative intracellular regulator, i.e. the CCM complex (Fig. 5D).

## DISCUSSION

The control of integrins activity is of critical importance in vivo and an impaired ability to activate or inactivate integrins is associated with human diseases (Winograd-Katz et al., 2014). It has been shown that the shear stress sensed at cell–cell junctions activates downstream pathways at cell–matrix adhesions where integrins play a key role (Collins et al., 2012). Here we demonstrate that the control of β1 integrin activation by CCM complex is crucial for the morphological response of endothelial cells to shear stress. One of the most significant findings to emerge from the present study is the existence of a balance between antagonistic extra- and intracellular signals, i.e. shear stress and CCM complex. This balance operates to permit the appropriate response of EC to flow (Fig. 5D).

### Control of β1 integrin activation by CCM complex is necessary for fine-tuned shear-stress response

Activation of β1 integrin by shear stress was previously reported (Jalali et al., 2001) and the involvement of this integrin in the mechanical response of EC to shear stress or cyclic mechanical strain has been demonstrated using blocking β1 integrin peptide or antibody (Thodeti et al., 2009).

In the present study we demonstrate that the depletion of β1 integrin or overexpression of its inhibitory complex CCM blocks remodeling of the actin cytoskeleton, EC elongation and orientation towards the direction of flow. It was previously shown that the CCM complex is crucial to control the mechanics of important endothelial functions such as VE-cadherin-mediated cell-to-cell or integrin-dependent cell–matrix adhesions as well as ECM remodeling (Faurobert et al., 2013). The relation between CCM complex disturbance and a defect in endothelial cell flow response was recently suggested by Mleynek et al. (Mleynek et al., 2014). Here we extend our knowledge by demonstrating the fundamental role of CCM complex in regulating the shear-stress-induced mechanical response of the EC. Indeed, our findings provide the first strong evidence that the β1 integrin-dependent response to shear stress is controlled by the CCM complex. We previously showed that β1 integrin is overactivated upon CCM complex loss (Faurobert et al., 2013). Here, we demonstrate that CCM complex depletion leads to an elongated “shear-stress-like” phenotype even in the absence of flow whereas the high levels of CCM proteins in the cell line EA.hy926 prevent the appearance of this phenotype upon a wide range of hemodynamic shear forces. This range spans over values reported for venous capillaries (below 1 Pa) (Koutsiaris et al., 2007) to that for arteries (between 2 and 6 Pa) (Kroll et al., 1996). In other words, CCM proteins antagonize the effect of shear stress to limit the EC mechanical response to flow. We used a shear stress of 2 Pa to study the effect of CCM2 silencing on cell orientation and elongation in HUVEC and EA.hy926 because lower shear stress leads to milder changes in cell morphology. Selecting a 2 Pa shear stress allowed us to detect clear differences and to clarify the mechanism. It is likely that inhibition of the cell response by CCM proteins is even stronger at lower pressure as the counterbalancing positive effect of shear stress on β1 integrin activation is reduced. Further studies using a range of shear stresses found in venous capillary should ascertain this point. Our assumption is that *in vivo*, the inhibitory effect of CCM proteins might impact on the EC response mainly at low hemodynamic forces. Indeed, the response of the EC to high shear stress (2 Pa) is similar when the CCM complex is present (naive HUVEC) or absent (CCM2-depleted EA.hy926) suggesting that, at this range of pressure, the inhibitory action of the CCM complex is overcome by the stimulatory action of flow. Therefore, either in physiological or upon CCM depletion conditions, arterial EC would always undergo a β1 integrin-dependent mechanical response necessary for them to stiffen and resist to external forces. At the low hemodynamic force found in venous capillaries, we propose that in physiological conditions, the CCM complex is necessary for maintaining β1 integrin in a low activation state. The loss of this complex in CCM pathology would lead to an abnormally high inside-out activation of β1 integrin in venous capillaries and to inappropriate cellular response. Previously published results shows that same level of shear stress can induce different responses depending on EC type (Pang et al., 2005; Ye et al., 2014), suggesting that there may be an evolutionary advantage to respond or to resist to shear stress depending on location of EC in different organs. This different EC responsiveness may arise from different level of expression of CCM proteins and ICAP-1 as observed in HUVEC and EA.hy926. Comparing the level of CCM proteins in the cerebral microvasculature with the levels of CCM proteins in microvessels of other tissues might help to understand why CCM lesion formation occurs predominantly in the brain.

Experiments on β1 integrin-deficient animals have shown that β1 integrin is a critical regulator of vascular physiology and its absence within the endothelium during development is lethal (Lei et al., 2008). In addition to regulating the organization of the actin cytoskeleton, activation of integrins by flow has been shown to modulate gene expression and cell fate. As such, shear stress activates NF-kB-dependent inflammatory response through the integrin-p38 MAPK signaling cascade (Orr et al., 2005; Tzima et al., 2005) in EC. Similarly, stimulation of the IL8 gene expression in EC under shear stress is dependent on β1 and β3 integrins and on the actin cytoskeleton (Cheng et al., 2007). Therefore, mechanisms regulating β1 integrin activation must have profound effects on EC physiology and dysregulation of β1 integrin signaling could lead to vascular diseases. Interestingly, loss of α1β1 integrin by genetic deficiency or its blocking by antibody inhibited the flow-mediated dilation of mouse mesenteric arteries (Loufrani et al., 2008). This suggests that β1 integrin signaling is involved in vessel dilation in response to shear stress. We hypothesize that overactivation of β1 integrin in response to shear stress could be involved in the excessive dilation of CCM-depleted capillaries as observed in human CCM lesions. This plausible model, which would explain why the CCM complex loss affects only low-flow blood vessels, has now to be tested *in vivo*.

## MATERIALS AND METHODS

### Cell culture and transfection

EA.hy926 cells, obtained from the ATCC (Manassas, USA) were cultured in Dulbecco modified Eagle's medium (DMEM, Invitrogen, France), supplemented with 10% fetal bovine serum, Hepes buffer (10 mM, PAA), glutamine (2 mM, PAA) and antibiotics. Passages 18–26 of EA.hy926 cell line were used. HUVEC cells were purchased from PromoCell, plated on fibronectin coated flasks and cultured in EC growth medium (PromoCell) supplemented with antibiotics. Only early passages of HUVEC (between 2 and 4) were used. For β1 integrin silencing, HUVEC (1.5×10^6^ cells) were transfected three times at 24 h interval with the β1 siRNA (smart pool siGenome, Dharmacon) or CT siRNA (AGG-UAG-UGU-AAU-CGC-CUU-G) at concentration 20 nM by using 45 µl Lipofectamine RNAi max (Invitrogen) according to the manufacturer's instructions. Cells were used the day after the third round of transfection. Silencing efficiency of β1 siRNA was determined by western blot (Fig. 2E) and by real-time PCR (99±0.5%, N = 3, not shown). For CCM2 silencing, EA.hy926 cells were transfected three times at 24 h interval and HUVEC cells were transfected two times as described previously (Faurobert et al., 2013). Silencing efficiency of CCM2 siRNA was determined by western blot (Fig. 3D, Fig. 5B) and by real-time PCR (99±0.4% in EA.hy926 cell line and 95±0.8% in HUVEC, N = 3, not shown).

### Application of unidirectional laminar shear stress

Endothelial cells were plated to fibronectin-coated slides (10 µg/mL) and confluent monolayers were exposed to flow in a closed circulating system for 5 or 18 h or incubated under static conditions, in all cases at 37°C with 5% CO_2_. For flow experiments, we used a parallel plate flow chamber, which was developed to study the effect of wide range of shear stress as described previously (Chotard-Ghodsnia et al., 2002; Macek Jilkova et al., 2013). Briefly, the flow chamber consists in two stainless steel parts enclosing a pair of parallel glass plates to allow observation by microscopy. The flow channel has a rectangular cross section with the following dimensions: length: l = 55 mm; width: w = 14 mm; height: h = 0.13 mm. A constant flow rate (Q) was imposed to the flow chamber using a peristaltic pump. The dynamic viscosity of circulating medium at 37°C was μ = 0.0007 Pa.s. The flow in the described chamber of constant rectangular cross-section is considered as two-dimensional (*h<<w*) and nearly unidirectional providing a fully developed flow. The Reynolds number, defined as *R*e = *ρQ*_in_/μ*w*, is small enough to assume laminar flow. Thus, the wall shear stress *τ* can be considered as constant along the flow chamber and is given by: τ = 6*μQ/wh*^2^. The constant shear stress applied to cells varies from 0.05–3.7 *Pa* (0.5 to 37 dynes/cm^2^), which corresponds to the physiological shear stress occurring in different parts of the human bloodstream. To confirm our results in a different flow setup, we also used a commercially available flow channel (μ-Slide I 0.4 Luer, IBIDI) coated by fibronectin (10 µg/mL).

### Analysis of cell alignment and shape

To perform morphometric analyses, digitized images from 6 different randomly chosen areas per each of 4–5 independent experiments for either static or shear stress conditions were acquired using the Axio Observer Z1 microscope (Zeiss), and a MicroMAX N/B camera (Princeton Instruments). Orientation of actin fibers relative to the flow direction was determined with ImageJ software. Cell orientation was assessed as an angle between the long axis of the cell and the direction of flow. 0° indicates perfect alignment with respect to the flow direction while a value of 45° indicates no alignment. The elliptical form factor was determined as the ratio of cell's length to its breadth, 1 indicates circle i.e. no elongation. Cells were analyzed with the MetaMorph software (n≥500).

### Immunocytochemistry and confocal fluorescence microscopy

Cell monolayers on glass slides were washed briefly with PBS containing calcium and magnesium. Cells were fixed with 2% paraformaldehyde and, if needed, permeabilized with 0.5% Triton X-100. After fixation, the cell monolayer was blocked with 10% normal goat serum in PBS. Monoclonal rat anti human activated-state-specific β1-integrin antibody (β1 integrin; Clone:9EG7; BD Biosciences, cat. no.: 553715, dilution: 1/200) was applied on non-permeabilized cells. Monoclonal mouse anti-human zyxin (Santa Cruz, dilution: 1/200) and monoclonal mouse anti-human paxillin (Upstate Biotechnology, dilution: 1/200) antibodies were used on permeabilized cells. Human VE-cadherin antibody was applied as described previously (Rival et al., 1996). Cells were incubated with primary antibody for 1 h. Goat anti-mouse Alexa 488 and Alexa 546 or goat anti-rat Alexa 488 IgG (Invitrogen) was applied for 30 min. To visualize F-actin, Texas Red phalloidin (Molecular Probes, dilution: 1/500) was applied for 30 min. Fluorescent images of basal cell surfaces were obtained using LSM710 NLO confocal microscope (Carl Zeiss).

### Measurement of cell surface ICAM-1 by flow cytometry

After having or not exposed ECs to shear stress, they were washed with PBS containing calcium and magnesium, and detached by incubation with Accutase solution (Sigma) for 2 min at 37°C. ECs were then centrifuged and gently re-suspended in medium containing FBS. The primary antibodies for ICAM-1 (Languino et al., 1995) or control IgG were added and the cell suspension was incubated at 4°C for 60 min. Secondary antibody, Alexa 488 goat anti-mouse (Invitrogen), was incubated with cells for 30 min and the fluorescence signal was analyzed by flow cytometry with an Accuri C6 flow cytometer using CflowPlus software (AccuriCytometers).

### Measurement of β1 integrin activation by flow cytometry

ECs were grown at confluency and harvested by trypsin treatment. They were incubated on ice for 15 min with or without 0.5 mM MnCl_2_. HUTS-4 antibody was added to the cell suspension at 5 µg/ml and incubated for 30 min on ice. Cells were washed and labeled with fluorophore-conjugated secondary antibody (AlexaFluor 647 goat anti-mouse). After final wash, cells were fixed in 4% PFA for 10 min on ice, washed in PBS, and then analyzed using a BDLSRII flow cytometer. The integrin index activation was calculated as the mean fluorescence intensity of HUTS-4 staining (active β1-integrin) divided by the mean fluorescence of HUTS-4 staining in presence of MnCl_2_ (total β1-integrin). Results were expressed as percentage of activated β1 integrin.

### Western blot analysis

Cells were lysed in Laemmli buffer, run on SDS-PAGE and transferred on PVDF membranes. The following antibodies were used: CCM2 (rabbit pAb anti-human, Acris AP26022PU-N, dilution: 1/1000); CCM1 (rabbit pAb anti-human (Faurobert el al., 2013), dilution: 1/1000); ICAP-1 (rabbit pAb anti-human (Millon-Frémillon et al., 2008), dilution: 1/1000); β1 integrin (rabbit pAb anti-human (Martel et al., 2001), dilution: 1/500).

Immunological detection was achieved with HRP-conjugated secondary antibody. Peroxidase activity was visualized by ECL (Bio-Rad) using a ChemiDoc MP imaging system (Bio-Rad). Densitometric quantification of the bands was performed using the Image Lab program (Bio-Rad). Protein levels were normalized to the level of actin or per total protein.

### Quantitative RT-PCR (qPCR)

Total RNA was extracted from cells using NucleoSpin RNA II kit (Macherey-Nagel) according to the manufacturer's instructions. RNA (1 µg) was reverse transcribed using SuperScript VILO kit (Life Technologies). Quantitative real-time PCR was performed with GoTaqR QPCR Master Mix (Promega) in a 25 µl reaction on a C-1000 Touch Thermal Cycler (BioRad). Product sizes were controlled by DNA gel electrophoresis and the melt curves were evaluated using the BioRad CFX Manager. Ct-values were determined with the same software, and normalization was done with the house keeping genes ATP50 and RPLO, yielding very similar results. The following primer pairs were used: CCM1 (For 5′-gaagcgcctgtgaaggagattc-3′, Rev 5′-acaatatgcgagtggcctcaac-3′); CCM2 (For 5′-cctgcacagcgatgactct-3′, Rev 5′-accacccacatccacagatt-3′); β1 (For 5′-acatctgtgaatgtgaatgcc-3′, Rev 5′-caatgtctaccaacacgcc-3′); ATP50 (For 5′-attgaaggtcgctatgccacag-3′, Rev 5′-aacagaagcagccactttggg-3′); RPLO (For 5′-tgctcaacatctcccccttctc-3′, Rev 5′-actggcaacattgcggacac-3′).

### Statistics

Data were analyzed using the SigmaStat statistical software. One-way ANOVA followed by multiple comparisons with Holm–Sidak test was used. For direct comparisons, an unpaired Student's *t* test was used. All values are presented as means ± SE.

### Abbreviations

CCM: Cerebral Cavernous Malformation; EC: Endothelial Cell; ECM: ExtraCellular Matrix; HUVEC: Human Umbilical Vein Endothelial Cell; VE-cadherin: Vascular-Endothelial Cadherin.
